# *Lactobacillus* Biofilms Influence Anti-*Candida* Activity

**DOI:** 10.3389/fmicb.2021.750368

**Published:** 2021-10-29

**Authors:** Carola Parolin, Vanessa Croatti, Luca Laghi, Barbara Giordani, Maria Rosaria Tondi, Priscilla Romina De Gregorio, Claudio Foschi, Beatrice Vitali

**Affiliations:** ^1^Department of Pharmacy and Biotechnology, Alma Mater Studiorum – University of Bologna, Bologna, Italy; ^2^Department of Agricultural and Food Sciences, Centre of Foodomics, Alma Mater Studiorum – University of Bologna, Bologna, Italy; ^3^National Institute of Geophysics and Volcanology, Bologna, Italy; ^4^Centro de Referencia para Lactobacilos (CERELA)-CONICET, San Miguel de Tucumán, Argentina; ^5^Department of Diagnostic and Specialty Medicine, Section of Microbiology, Alma Mater Studiorum – University of Bologna, Bologna, Italy

**Keywords:** *Lactobacillus*, *Candida*, biofilm, metabolome, vaginal microbiota, health benefits

## Abstract

Lactobacilli are the dominant members of the healthy human vaginal microbiota and represent the first defense line from pathogen infection, including vulvovaginal candidiasis. Biofilm is the predominant microbial growth form in nature, and the formation of biofilms inside the human body has important implications in health and disease. In particular, the formation of biofilm by members of the human resident microbiota is desirable, as it can improve microbial persistence and influence functionality. In the present study, we investigated the capability of 16 vaginal *Lactobacillus* strains (belonging to *Lactobacillus crispatus*, *Lactobacillus gasseri, Lactobacillus vaginalis*, and *Lactobacillus plantarum* species) to form biofilms, and we correlated their mode of growth to anti-*Candida* activity. *L. plantarum* strains were the best biofilm producers, and high variability was registered in the level of biofilm formation among *L. crispatus* and *L. gasseri* strains. Culture supernatants derived from *Lactobacillus* biofilm and planktonic growth were tested toward a panel of *Candida* clinical isolates (*Candida albicans, Candida glabrata, Candida lusitaniae*, *Candida tropicalis*, *Candida krusei*, and *Candida parapsilosis*) and their metabolome assessed by ^1^H-NMR. *L. crispatus* and *L. plantarum* strains exhibited the best fungistatic profile, and biofilms enhanced their anti-*Candida* activity; on the contrary, *L. gasseri* strains were more effective when grown in a planktonic mode. Biofilm/planktonic mode of growth also affects *Lactobacillus* metabolism, mainly influencing nitrogen and amino acid pathways, and anti-*Candida* activity is instead strictly related to carbohydrate metabolism. The present study underlined the strict interdependence between microbial mode of growth, metabolism, and functional properties. Biofilm formation by members of the healthy human microbiota represents a crucial issue in the field of microbial physiology and host–microbiota interactions, beyond supporting the development of new antimycotic strategies based on probiotics grown in adherence.

## Introduction

Members of the *Lactobacillus* genus are abundant and predominant in the vaginal niche of healthy women of reproductive age, reaching a concentration of 10^7^ cfu/ml of vaginal sample, and 80% of the whole microbial content (Rönnqvist et al., 2006; Ceccarani et al., 2019). Most healthy women show the dominance of one species of *Lactobacillus*, and *Lactobacillus crispatus*, *Lactobacillus iners*, *Lactobacillus jensenii*, and *Lactobacillus gasseri* are the most frequent (Ravel et al., 2011). It is widely demonstrated that vaginal lactobacilli are involved in maintaining the state of vaginal eubiosis, preventing the colonization of genital, and sexually transmitted pathogens. Indeed, it has been reported that various vaginal *Lactobacillus* strains can interfere with *Candida* spp. overgrowth and reduce vulvovaginal candidiasis occurrence (Parolin et al., 2015; De Gregorio et al., 2019; Namarta et al., 2020). Anti-*Candida* activity of lactobacilli can be mediated through multiple mechanisms, including pH lowering, secretion of effective compounds, and impairment of fungal adhesion (Parolin et al., 2015, 2021; Calonghi et al., 2017; Abruzzo et al., 2018, 2021; De Gregorio et al., 2019).

Microorganisms’ growth can occur in two different forms: planktonic and biofilm, and the latter is the predominant form in nature (Rieu et al., 2014). The biofilm mode of growth is characterized by high cell density and secretion of a polymeric matrix, in which microorganisms are immersed (Flemming and Wingender, 2010). The formation of microbial biofilms inside the human body has important implications in health and disease: for example, pathogens’ biofilms generally show increased resistance to antibiotics, making their elimination challenging. On the other hand, the formation of biofilm by members of the human resident microbiota is desirable, as it can improve microbial persistence and favor functionality (Li et al., 2018). The planktonic growth or the alternative establishment of a sessile biofilm by a microbial species implies the remodeling of physiological and metabolic pathways, influencing in turn functional properties of probiotics. For some *Lactobacillus* species, it has been reported that biofilm mode of life can improve antagonistic effects toward bacterial pathogens and anti-inflammatory and immunomodulatory properties (Rieu et al., 2014; Aoudia et al., 2016; Liu et al., 2021), although few studies have investigated this issue till now.

In the present manuscript, 16 vaginal *Lactobacillus* strains, belonging to *L. crispatus*, *L. gasseri, Lactobacillus vaginalis*, and *Lactobacillus plantarum* species, were tested for their *in vitro* capacity to form biofilms. Culture supernatants derived from planktonic and biofilm growth were tested against a panel of *Candida* clinical isolates (*Candida albicans, Candida glabrata, Candida lusitaniae*, *Candida tropicalis*, *Candida krusei*, and *Candida parapsilosis*). Anti-*Candida* activity was then correlated with lactobacilli culture supernatants metabolome, assessed by ^1^H-NMR.

## Materials and Methods

### Microorganisms and Culture Conditions

All the 16 *Lactobacillus* strains included in this study were previously isolated from vaginal swabs of healthy premenopausal Caucasian women, following the protocol approved by the Ethics Committee of the University of Bologna, Bologna, Italy (52/2014/U/Tess) (Parolin et al., 2015). *Lactobacillus* strains are listed in Table 1. Lactobacilli were routinely cultured in de Man, Rogosa, and Sharpe (MRS) broth (Difco, Detroit, MI, United States) supplemented with 0.05% L-cysteine (Merck, Milan, Italy), at 37°C and in anaerobiosis. Anaerobic conditions were achieved by using jars containing GasPak^TM^ (GasPak^TM^ EZ Anaerobe Container System, Becton, Dickinson and Co., Sparks, MD, United States). Before the experiments, each strain was transferred from the frozen stock to MRS broth and allowed to grow for 24 h and then subcultured in the same medium and conditions for an additional 24 h.

**TABLE 1:** List of microorganisms used in the present manuscript and origin (VVC: vulvovaginal candidiasis).

Species	Strain denomination	Origin
***Lactobacillus* strains**		

*L. crispatus*	BC1	Healthy human vagina
*L. crispatus*	BC3	Healthy human vagina
*L. crispatus*	BC4	Healthy human vagina
*L. crispatus*	BC5	Healthy human vagina
*L. crispatus*	BC6	Healthy human vagina
*L. crispatus*	BC7	Healthy human vagina
*L. gasseri*	BC9	Healthy human vagina
*L. gasseri*	BC10	Healthy human vagina
*L. gasseri*	BC11	Healthy human vagina
*L. gasseri*	BC12	Healthy human vagina
*L. gasseri*	BC13	Healthy human vagina
*L. gasseri*	BC14	Healthy human vagina
*L. vaginalis*	BC16	Healthy human vagina
*L. vaginalis*	BC17	Healthy human vagina
*L. plantarum*	BC18	Healthy human vagina
*L. plantarum*	BC19	Healthy human vagina
***Candida* strains**		
*C. albicans*	SO1	VVC human vagina
*C. albicans*	SO2	VVC human vagina
*C. glabrata*	SO17	VVC human vagina
*C. glabrata*	SO18	VVC human vagina
*C. lusitaniae*	SO22	VVC human vagina
*C. tropicalis*	SO24	VVC human vagina
*C. krusei*	SO26	VVC human vagina
*C. parapsilosis*	SO27	VVC human vagina

The eight *Candida* strains used in the present study belong to a broad collection of yeasts isolated from vaginal swabs of premenopausal, VVC-affected women during routine diagnostic procedures at the “Microbiology Laboratory” in Sant’Orsola-Malpighi University Hospital of Bologna, Bologna, Italy. All the clinical isolates were coded to assure full anonymousness and are listed in Table 1 (Oliver et al., 2020; Parolin et al., 2021). *Candida* strains were grown aerobically in Sabouraud dextrose (SD) medium (Difco) at 35°C in aerobiosis.

### *Lactobacillus* Biofilm Formation Assay

To assess *Lactobacillus* strains’ capability to form biofilms, bacterial pellets from the second subculture were washed in sterile saline and resuspended in MRS broth to a final concentration of 10^7^ colony-forming units (cfu) per milliliter. The bacterial suspension was inoculated in polystyrene, U-bottom 96-well plates (0.2 ml per well) and allowed to grow for 72 h at 37°C, in anaerobiosis. At the end of the incubation, biofilm formation was evaluated by crystal violet staining, following the protocol described by Leccese Terraf et al. (2014). Briefly, culture supernatant was discarded and wells were gently washed with phosphate buffer, pH 7.4. Adherent biofilm was stained with 0.1% (w/v) crystal violet (CV) aqueous solution for 30 min and then washed with distilled water. Plates were dried overnight at room temperature, and the CV bound to the adherent biofilm was solubilized in 30% (v/v) acetic acid. CV absorbance was measured at 595 nm with an Enspire multiplate reader (Perkin Elmer, United States). Each strain was assayed in at least three independent experiments, each with four replicates. Additionally, a sterile culture medium was always included as negative control.

### Preparation of Cell-Free Supernatants From *Lactobacillus* Planktonic and Biofilm Cultures

*Lactobacillus* strains were grown in planktonic and biofilm forms and culture supernatants collected. 10^7^ cfu/ml bacterial suspensions were prepared as described above and inoculated in sterile glass tubes (10 ml, planktonic form) or polystyrene six-well plates (4 ml per well, biofilm form). Planktonic (pk) and biofilm (bf) samples were incubated for 72 h at 37°C, in anaerobiosis. Afterward, culture supernatants were recovered, centrifuged (2,750 × *g*, 10 min) and filtered through a 0.22-μm membrane filter to obtain cell-free supernatants (CFS). CFS obtained from planktonic (pk-CFS) and biofilm (bf-CFS) cultures were stored at −20°C until their use.

### Metabolomics of *Lactobacillus* Cell-Free Supernatants

After centrifugation at 18,630 × *g* for 10 min at 4°C, 0.7 ml of pk-CFS and bf-CFS samples were added to 0.1 ml of a D2O solution of 3-(trimethylsilyl)-propionic-2,2,3,3-d4 acid sodium salt (TSP) 10 mM, set to pH 7.0 by means of a 1 M phosphate buffer. The solution contained also NaN_3_ 2 mM, to avoid microorganisms’ proliferation. ^1^H-NMR spectra were recorded with an AVANCE III spectrometer (Bruker, Milan, IT, United States) at 298 K operating at a frequency of 600.13 MHz. To reduce broad signals caused by slowly tumbling molecules, a CPMG filter of 400 echoes, separated by an echo time of 400 μs, was applied. Water residual signal was reduced by presaturation. The signals were assigned by comparing their chemical shift and multiplicity with Chenomx software data bank (Chenomx Inc., Canada, ver 11.05) (Laghi et al., 2014). Metabolite concentrations were calculated and reported as differences with respect to MRS medium.

### Anti-*Candida* Activity of *Lactobacillus* Supernatants

Planktonic and biofilm supernatants anti-*Candida* activity was tested by microdilution assay, following EUCAST guidelines (EUCAST Committee, 2020). Briefly, stock *Candida* suspensions prepared in sterile water at an absorbance (measured at 600 nm) of 0.008–0.137 were diluted 1:10 in RPMI 1640 medium buffered to pH 7.0 with 0.165 M MOPS (morpholinepropanesulfonic acid buffer) and added with 2% glucose. Final yeast suspensions, corresponding to 1–5 × 10^5^ cfu/ml, were inoculated in flat-bottomed 96-well plates (0.1 ml per well) and added with the same volume of each *Lactobacillus* pk-CFS and bf-CFS. Positive growth control wells contained 0.1 ml of *Candida* suspension added with the same volume of sterile MRS medium. The plates were incubated at 35°C for 24 h; afterward, *Candida* growth was evaluated by reading the absorbance at 530 nm with an Enspire multiplate reader. *Candida* growth inhibition was calculated relative to the absorbance of the corresponding positive controls. A subset of *Lactobacillus* pk-CFS and bf-CFS was tested for anti-*Candida* activity in simulated vaginal fluid (SVF), pH 4.2, by microdilution assay, following the method described above. In brief, *Candida* suspensions were diluted 1:10 in SVF, composed of 3.51 g/L NaCl, 1.40 g/L KOH, 0.222 g/L Ca(OH)_2_, 0.018 g/L bovine serum albumin, 2 g/L lactic acid, 1 g/L acetic acid, 0.16 g/L glycerol, 0.4 g/L urea, and 5 g/L glucose. The pH was adjusted to 4.2 with HCl 0.1 N and the solution was filtered through a 0.22-μm membrane filter. CFS obtained from *L. crispatus* BC1, *L. crispatus* BC6, *L. gasseri* BC11, *L. gasseri* BC12, *L. vaginalis* BC17, and *L. plantarum* BC19 were tested.

Cell-free supernatants fungicidal activity was assessed by spotting 20 μl of active samples onto SD agar plates, then incubated at 35°C for 24/48 h. *Candida* viability was determined by the ability of forming colonies.

### Data Analysis and Statistics

Data were analyzed by MatLab software (R2020b version 9.9.0.1524771, The MathWorks Inc., Natick, MA, United States) with the Statistics and Machine Learning Toolbox. Cluster analysis was performed by means of t-Distributed Stochastic Neighbor Embedding method (Van der Maaten and Hinton, 2008). Differences in biofilm formation were assessed by Kruskal–Wallis test followed by Tukey-Kramer test, and differences in metabolite concentrations were assessed by Wilcoxon rank test. Enrichment analysis was performed on significantly different metabolites, by using MetaboAnalyst web resource^1^. Differences were deemed significant for *p* < 0.05.

## Results

### Ability of Vaginal Lactobacilli to Form Biofilm

**FIGURE 1:**
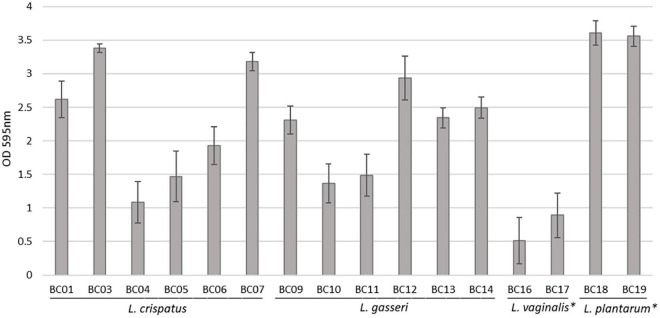
Biofilm formation by *Lactobacillus* strains, evaluated by crystal violet staining (average ± st. dev.). *p < 0.05 selected species vs. other *Lactobacillus* species.

Sixteen *Lactobacillus* strains isolated from vaginal swabs were tested for their capability to form biofilm on an abiotic polystyrene surface (Figure 1). In general, all vaginal *Lactobacillus* used in this study can form biofilm, although at different amounts (*p* < 0.05). The strongest biofilm producers were *L. plantarum* BC18 and BC19 strains (*p* < 0.05), while *L. vaginalis* BC16 and BC17 strains were the weakest (*p* < 0.05). Within *L. crispatus* and *L. gasseri* species, very high variability was observed. In particular, among *L. crispatus*, BC3 and BC7 exhibited the highest biofilm production; for *L. gasseri*, BC12 was the best producer.

### Anti-*Candida* Activity of Lactobacilli in Planktonic/Biofilm Cultures

**FIGURE 2:**
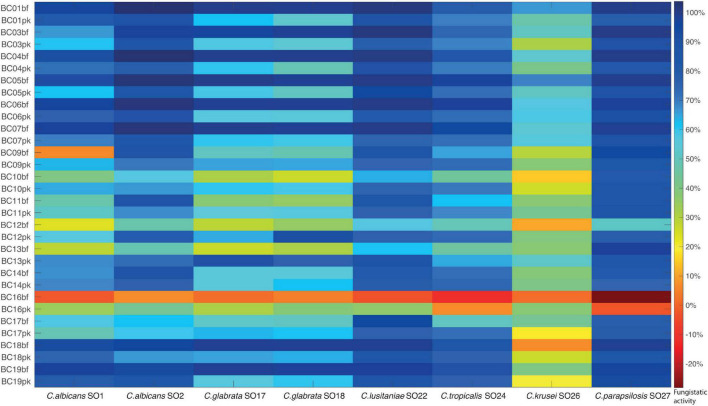
Fungistatic activity of *Lactobacillus* bf-CFS and pk-CFS toward clinical *Candida* isolates, tested following EUCAST guidelines (pH 7). OD 530 nm values were normalized for positive controls and data were expressed as inhibition (%) of *Candida* growth. Fungistatic activity color scale was reported (10% activity variation).

The fungistatic activity of bf-CFS and pk-CFS of vaginal lactobacilli was evaluated toward eight *Candida* clinical isolates, belonging to *C. albicans* and non-*albicans* species (Table 1). The anti-*Candida* activity was firstly assessed following EUCAST guidelines for antifungal susceptibility testing (EUCAST Committee, 2020), which imply RPMI supplemented with glucose and MOPS, pH 7. Overall, the most sensitive *Candida* isolates were *C. albicans* SO2, *C. lusitaniae* SO22, and *C. parapsilosis* SO27, whereas *C. krusei* SO26 was the most resistant to *Lactobacillus* CFS (Figure 2). bf-CFS from *L. crispatus* and *L. plantarum* strains showed the highest fungistatic activity toward all *Candida* isolates, exhibiting the best anti-*Candida* profile. In detail, *L. crispatus* and *L. plantarum* bf-CFS showed higher values of *Candida* growth inhibition than the respective pk-CFS: bf-CFS almost completely inhibited (over 95% growth inhibition) *C. albicans* SO2, *C. glabrata* SO17, *C. glabrata* SO18, *C. lusitaniae* SO22, and *C. parapsilosis* SO27 growth, and strongly inhibited (over 65% inhibition) *C. albicans* SO1 and *C. tropicalis* SO24. The corresponding pk-CFS showed inhibition rates ranging from 50 to 90% toward the same *Candida* strains. On the contrary, most *L. gasseri* strains exerted higher anti-*Candida* activity when they were cultured in planktonic mode rather than in biofilm, and great variability among *Candida* isolates’ inhibition was registered. Overall, *L. gasseri* pk-CFS were mostly active toward *C. lusitaniae* SO22 and *C. parapsilosis* SO27, and to a lesser extent on *C. albicans* SO2. Intermediate anti-*Candida* activity was shown on *C. glabrata* SO17, *C. glabrata* SO18, and *C. tropicalis* SO24. *L. vaginalis* BC16 bf-CFS and pk-CFS showed the lowest fungistatic activity against all *Candida* isolates, exhibiting the worst anti-*Candida* profile. Overall, CFS were not fungicidal, with the only exception of *L. crispatus* bf-CFS toward *C. parapsilosis* SO27 and *L. plantarum* bf-CFS toward *C. lusitaniae* SO22.

**FIGURE 3:**
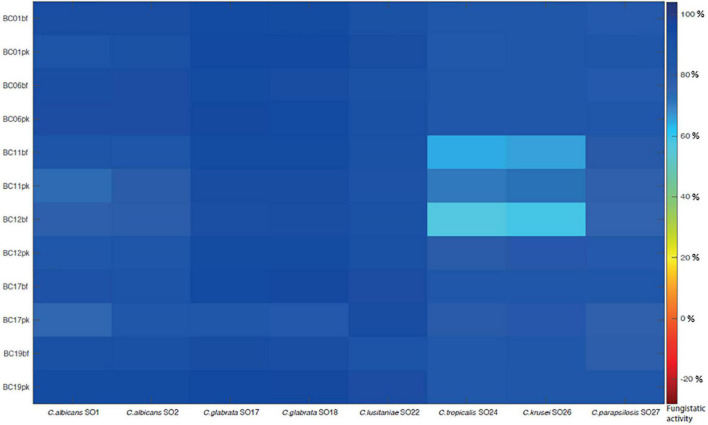
Fungistatic activity of *L. crispatus* BC1, *L. crispatus* BC6, *L. gasseri* BC11, *L. gasseri* BC12, *L. vaginalis* BC17, and *L. plantarum* BC19 bf-CFS and pk-CFS toward clinical *Candida* isolates, tested in simulated vaginal fluid, pH 4.2. OD 530 nm values were normalized for positive controls and data were expressed as inhibition (%) of *Candida* growth. Fungistatic activity color scale was reported.

In order to mimic the physiological condition typically found in the vaginal environment, the fungistatic activity of CFS was also tested in SVF, pH 4.2. A subset of active CFS was selected (i.e., CFS obtained from *L. crispatus* BC1, *L. crispatus* BC6, *L. gasseri* BC11, *L. gasseri* BC12, *L. vaginalis* BC17, and *L. plantarum* BC19) and tested toward the panel of *Candida* isolates (Figure 3). Also in these experimental conditions, CFS showed a remarkable anti-*Candida* activity; interestingly, *Lactobacillus* CFS were highly active also toward *C. glabrata* SO17, *C. glabrata* SO18, and *C. krusei* SO26 (over 60% inhibition). Overall, acidic pH of SVF enhanced *Candida* growth inhibition by CFS, especially for pk-CFS.

**FIGURE 4:**
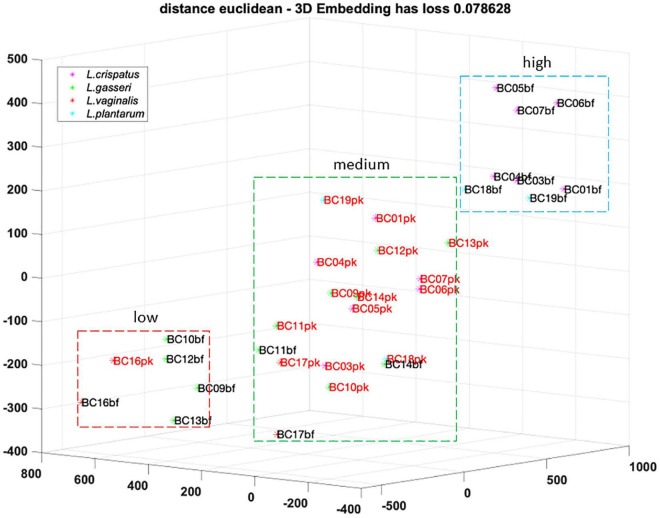
t-SNE cluster analysis on CFS fungistatic activity dataset. Three groups were identified, corresponding to low, medium, and high activity. Samples derived from biofilm and planktonic cultures were signed in black and red, respectively.

To better understand the analogies of CFS activities toward *Candida* isolates, a cluster analysis was performed using the t-SNE method on data obtained following EUCAST guidelines (Figure 4). Considering the position of samples in the 3D map, we identified three groups of CFS defined as low, medium, and high activity. All *L. crispatus* and *L. plantarum* bf-CFS are grouped in the high-activity cluster, while pk-CFS from the same species and *L. gasseri* pk-CFS are grouped in the medium one. *L. gasseri* bf-CFS, except for *L. gasseri* BC11 and BC14 bf-CFS, are grouped with *L. vaginalis* BC16 CFS and demonstrated a low fungistatic activity.

### *Lactobacillus* Metabolome Analysis

**FIGURE 5:**
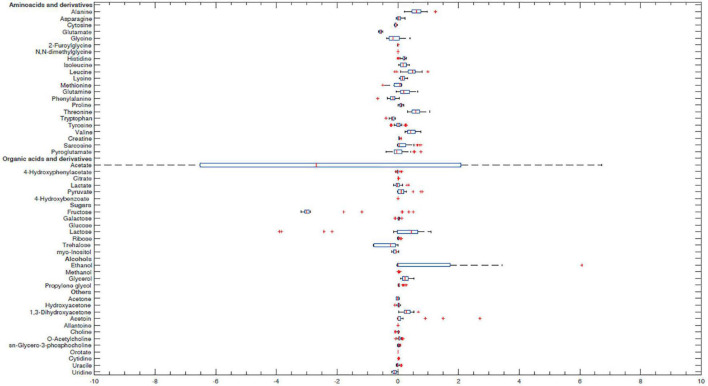
Box plot of *Lactobacillus* CFS metabolite concentrations. Concentrations were calculated as differences from MRS broth (mmol/L). Lines within the boxes indicate the median values of the samples. Each box represents the interquartile range (25–75th percentile). The extremes of the bars indicate the 10th and 90th percentiles, respectively. Outlier values are indicated (+). Glucose median and acetate interquartile range are not represented because they were out of the range considered.

In order to better characterize vaginal *Lactobacillus* CFS and bf-CFS and pk-CFS peculiarities, their metabolomic profiles were obtained by ^1^H-NMR. Fifty-one molecules belonging to the families of amino acids, organic acids, monosaccharides/disaccharides, ketones, and alcohols were identified (Figure 5), and differences were calculated with respect to MRS medium. All metabolites showed similar concentration median values, except for acetate and glucose. The same molecules also showed high variability among samples. We thus investigated the metabolites that mainly differentiate bf-CFS and pk-CFS samples, and we found out that the quantities of 17 molecules were significantly different between the two groups (Table 2). Among those, 10 amino acids (or derivatives) were identified, suggesting a deep involvement of this molecule category. Data were then subjected to overrepresentation analysis (Supplementary Figure 1), which highlighted that the growth form (bf vs. pk) mainly affects the nitrogen metabolism, and specifically several amino acidic pathways (serine, alanine, and lysine).

**TABLE 2:** List of metabolites significantly different between *Lactobacillus* bf-CFS and pk-CFS (*p* < 0.05) and concentrations, calculated as differences with respect to MRS medium [median value ± MAD (mmol/L)].

Metabolites	bf-CFS	pk-CFS
**Amino acids and derivatives**		
Alanine	0.7532 ± 0.1226	0.4625 ± 0.0935
Asparagine	0.0153 ± 0.0377	0.0558 ± 0.0415
Glutamate	−0.5813 ± 0.0343	−0.5487 ± 0.0210
Glutamine	0.2304 ± 0.0972	0.1152 ± 0.1133
2-Furoylglycine	0.0044 ± 0.0013	0.0003 ± 0.0037
Leucine	0.5522 ± 0.1429	0.4502 ± 0.0953
Lysine	0.1953 ± 0.0708	0.0982 ± 0.0380
Methionine	−0.1272 ± 0.2105	0.1092 ± 0.0117
Threonine	0.5409 ± 0.0835	0.6537 ± 0.1103
Sarcosine	0.2565 ± 0.2150	0.0209 ± 0.0204
**Organic acids**		
Citrate	0.0145 ± 0.0024	0.0051 ± 0.0020
Ribose	0.0277 ± 0.0072	0.0092 ± 0.0075
**Alcohols**		
Glycerol	0.1559 ± 0.0674	0.2926 ± 0.0900
**Others**		
Acetone	−0.0489 ± 0.0013	0.0250 ± 0.0081
1,3-Dihydroxyacetone	0.2195 ± 0.0592	0.3872 ± 0.1100
Acetoin	0.0034 ± 0.0062	0.0214 ± 0.0119
Allantoin	0.0080 ± 0.0011	0.0060 ± 0.0013

### Correlation of Anti-*Candida* Activity With Supernatant Metabolomes

**FIGURE 6:**
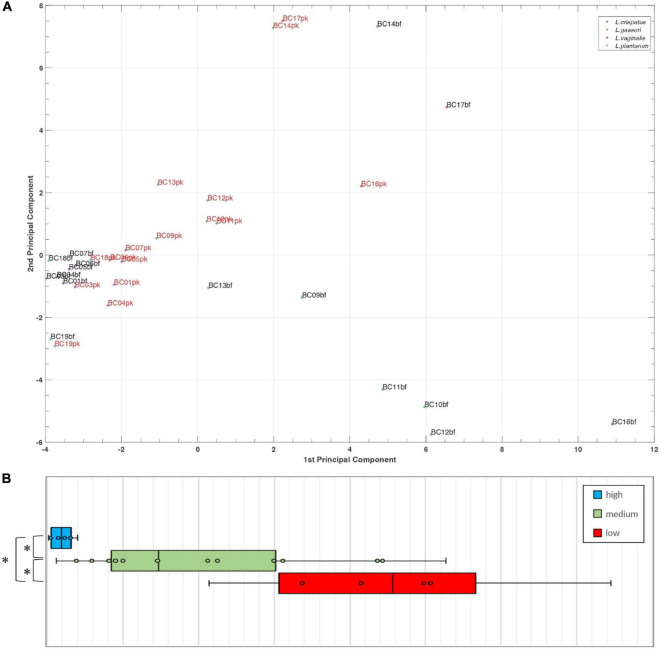
**(A)** Biplot of a PCA performed on *Lactobacillus* bf-CFS and pk-CFS metabolome and anti-*Candida* activity data. **(B)** Boxplot of CFS distribution on PC1 based on anti-*Candida* activity score. Lines within the boxes indicate the median values of the samples. Each box represents the interquartile range (25–75th percentile). The extremes of the bars indicate the 10th and 90th percentiles, respectively.

A principal component analysis (PCA) was performed for *Lactobacillus* bf-CFS and pk-CFS considering metabolomic and activity against *Candida* (Figure 6A). In the biplot, PC1 and PC2 represented the 44.3% of the total variance of the investigated samples (PC1 26.2%; PC2 18.1%). *Lactobacillus* CFS with different anti-*Candida* activity separated on PC1. In particular, *L. crispatus* and *L. plantarum* bf-CFS, which belong to the high anti-*Candida* activity group, were characterized by low values of PC1, while the low activity CFS samples showed high values of PC1 (Figure 6B). In order to identify the metabolites that mainly contribute to the anti-*Candida* activity of *Lactobacillus* CFS, we compared the metabolome of the active CFS to that of the less active ones. We found out that 18 molecule amounts were significantly different in the two groups (Table 3); most of them are represented by carbohydrates and amino acids. Enrichment analysis suggested a significant difference between the two CFS groups in the metabolism of sugars, mainly involving lactose and galactose pathways.

**TABLE 3:** List of metabolites significantly different between *Lactobacillus* CFS belonging to high anti-*Candida* activity group and low anti-*Candida* activity group (*p* < 0.05) and concentrations, calculated as differences with respect to MRS medium [median value ± MAD (mmol/L)].

Metabolites	High-activity CFS	Low-activity CFS
**Amino acids and derivatives**		
Glycine	−0.3130 ± 0.0406	−0.1280 ± 0.0770
Histidine	0.2398 ± 0.0091	0.0886 ± 0.0748
Isoleucine	0.1051 ± 0.0188	0.2457 ± 0.0427
Methionine	0.0747 ± 0.0113	−0.3244 ± 0.1486
Proline	0.0878 ± 0.0080	0.1261 ± 0.0143
Valine	0.3559 ± 0.0342	0.5096 ± 0.0647
Sarcosine	0.0570 ± 0.0155	0.5450 ± 0.1427
**Organic acids**		
Acetate	4.6614 ± 0.5951	−12.2848 ± 3.5317
Lactate	−0.0499 ± 0.0293	0.1147 ± 0.0749
Pyruvate	0.1285 ± 0.0199	0.0311 ± 0.0244
**Sugars**		
Fructose	−3.0765 ± 0.0353	−0.5228 ± 0.9609
Galactose	0.0436 ± 0.0171	0.0169 ± 0.0102
Glucose	−83.9781 ± 0.0512	−58.5522 ± 13.3528
Ribose	0.0266 ± 0.0017	0.0609 ± 0.0340
myo-Inositol	−0.0588 ± 0.0197	−0.1496 ± 0.0204
**Others**		
sn-Glycero-3-phosphocholine	0.0284 ± 0.0051	0.0083 ± 0.0072
Orotate	0.0059 ± 0.0015	−0.0005 ± 0.0008
Uracil	−0.0167 ± 0.0064	0.0527 ± 0.0253

## Discussion

The two different forms of microbial growth, i.e., planktonic and biofilm, differ for physiological and metabolic features; indeed, the establishment of a sessile culture by a microbial strain requires the remodeling of several molecular pathways involved in adhesion, secretion, and other cellular functions. Such modifications of the global metabolism can, in turn, influence the functional properties of probiotics, beyond favoring the permanence of probiotics inside the human body (Donlan and Costerton, 2002; Ventolini, 2015).

In this context, we first assessed the ability of *Lactobacillu*s strains of vaginal origin, belonging to *L. crispatus, L. gasseri, L. vaginalis*, and *L. plantarum* species, to form biofilm. We found out that *L. plantarum* species was the best biofilm producer, probably because of its adaptive features to niches (van den Nieuwboer et al., 2016). Indeed, *L. plantarum* is a versatile species able to adapt to different environmental niches and its genome, considered as one of the largest genome in lactic acid bacteria, encodes a huge repertoire of surface sugar, extracellular proteins, and regulatory peptide involved in the regulation of adherence (Kleerebezem et al., 2003; Sturme et al., 2005). Other authors previously reported that *L. plantarum* was able to produce a robust biofilm *in vitro*, resistant to environmental stresses (Kubota et al., 2008). Considering the most peculiar species of the vaginal microbiota, high variability was registered in the level of biofilm formation among *L. crispatus* and *L. gasseri* strains. Notably, *L. crispatus* BC3 biofilm was comparable to *L. plantarum* strains. Biofilm formation by *L. crispatus* and *L. gasseri* has been poorly investigated in the literature, and it has been reported that some *L. crispatus* strains of vaginal origin were not able to form biofilm and weakly autoaggregated (van der Veer et al., 2018), while Stivala et al. (2021) reported about vaginal *L. crispatus* and *L. gasseri* (and *L. rhamnosus*) strains as weak to moderate biofilm producers, with great variability.

From our results, the shift from a *Lactobacillus* planktonic culture to an adherent form implied major differences in the metabolism of nitrogen and amino acids, suggesting a key role of this molecule category in the formation of an adherent biomass, as already proposed by Liu et al. for *Lactobacillus paraplantarum* (Liu et al., 2021). In particular, alanine and lysine were produced/released at higher amounts by lactobacilli in the biofilm form compared to the planktonic one, whereas methionine was highly consumed by *Lactobacillus* in biofilm growth. These data support the finding that the methionine pathway is involved in the biofilm formation process, as proposed by Liu et al. (2018) in *L. paraplantarum*.

Although the anti-*Candida* activity of planktonic-derived *Lactobacillus* CFS has been reported and explored by several authors (Parolin et al., 2015; Wang et al., 2017; De Gregorio et al., 2019; Jang et al., 2019; Tortelli et al., 2020), no data are available on bf-CFS anti-*Candida* potential. In the present manuscript, both planktonic- and biofilm-derived CFS were tested against a broad spectrum of *Candida* clinical isolates, including *C. albicans* and *C. non-albicans* species. Overall, CFS did not exert fungistatic activity, with few exceptions. Fungistatic activity was tested in standard conditions following EUCAST protocol and validated in SVF. In accordance with results reported by Parolin et al. (2015), *C. krusei* resulted in being the most resistant isolate, although acidic conditions enhanced CFS fungistatic effect. *C. glabrata* isolates were susceptible to *Lactobacillus* CFS and notably their growth was almost abrogated by *L. crispatus* bf-CFS, although this species is often reported as resistant to antifungal substances (Rodrigues et al., 2014). Unlike previous results (Parolin et al., 2015) that were obtained with CFS from lactobacilli in exponential growth phase, *Lactobacillus* CFS from the stationary phase were active toward *C. parapsilosis*, pointing out the importance of growth phase and resulting metabolites on the anti-*Candida* activity. In addition, bf-CFS were more effective against *C. parapsilosis* than the respective pk-CFS.

*Lactobacillus* CFS that demonstrated high anti-*Candida* activity all derived from biofilm mode of growth and were produced by *L. crispatus* and *L. plantarum* strains; on the contrary, low-activity CFS were derived from *L. gasseri* (BC11–BC14) biofilm cultures and *L. vaginalis* BC16. This observation suggested that the anti-*Candida* activity of *L. crispatus* strains was enhanced when they were able to establish an adherent biofilm, while *L. gasseri* strains behaved in the opposite manner. This result, in turn, can offer new hints in the perspective of using *L. crispatus* or *L. gasseri* as probiotics to prevent vulvovaginal candidiasis or to restore the vaginal eubiosis status, also in the light of developing fourth-generation probiotics, based on biofilm-forming strains and encapsulation techniques (Salas-Jara et al., 2016). Peculiarly, *L. vaginalis* BC16, especially when grown in the biofilm mode, slightly stimulated *Candida* proliferation. This behavior correlated with *L. vaginalis* BC16 weak attitude to form an adherent biofilm, suggesting that its preferred mode of growth (i.e., planktonic) assured the best conditions to prevent from *Candida* infection, sustaining in turn the healthy status of the vaginal ecological niche.

In the present manuscript, we also sought for peculiarities in the metabolome of *Lactobacillus* CFS that demonstrated highly effective toward *Candida* clinical isolates. Highly active CFS differentiated from low active ones mostly for carbohydrates and amino acid quantification. Among carbohydrates, high anti-*Candida* activity was accompanied by increased consumption of glucose and fructose (corresponding to lower amounts), two well-known glycolytic fuel molecules. A marked consumption of glucose has already been correlated with *Lactobacillus* antimicrobial effect (Nardini et al., 2016), highlighting the mechanism of competition for nutrients between human beneficial microbes and pathogens as crucial. On the other hand, galactose concentration was higher in active CFS compared to non-active ones and lactose/galactose metabolism has been identified by overrepresentation analysis as a differential metabolic pathway between active and non-active CFS, denoting the preference of active *Lactobacillus* strains to utilize other carbon sources than galactose to sustain their growth.

Another recognized mechanism supporting the antimicrobial activity of lactobacilli is lactic acid production by fermentation and acidification (Boskey et al., 2001; O’Hanlon et al., 2013). In this regard, lower amount of lactate was quantified in active CFS compared to non-active ones, suggesting that the observed anti-*Candida* effect was not directly attributable to this organic acid. CFS endowed with high anti-*Candida* activity also showed higher amounts of pyruvate with respect to low-activity CFS, suggesting that the antifungal activity did not depend on a burst of the classical lactic fermentation pathway. In addition, active CFS contained high amounts of acetate, although *L. crispatus, L. gasseri*, and *L. plantarum* are recognized as homofermentative lactic acid bacteria. *Lactobacillus* CFS fungistatic activity was assessed in standard and acidic conditions, suggesting that the anti-*Candida* effect is not merely dependent on the acidic pH. Nevertheless, when CFS acted in an acidic environment, the anti-*Candida* activity was enhanced, pointing up the existence of a synergic activity among different components of the *Lactobacillus* supernatants and suggesting that the vaginal physiological environment could improve CFS anti-*Candida* potential.

To the best of our knowledge, the present study reports the anti-*Candida* activity of vaginal *Lactobacillus* grown in a sessile mode for the first time. Biofilm formation by lactobacilli represents a crucial aspect in the vaginal ecology, as this phenomenon can possibly influence endogenous microbiota persistence and functionality. Here, we demonstrated that *Lactobacillus* adherent growth strongly affects the anti-*Candida* potential and could in turn modulate *Candida* colonization of the vaginal niche. In particular, biofilm formation enhanced *L. crispatus* and *L. plantarum* fungistatic activity, while *L. gasseri* became more active when grown in a free form. Lactobacilli metabolism turned different between planktonic and biofilm forms, though the correlation between metabolism and functional properties demands more investigation. Further studies on lactobacilli biofilm formation in the vaginal niche will be approached to better understand its physiological role, also in the perspective to support the development of new antimycotic strategies based on probiotics grown in adherence.

## Data Availability Statement

The original contributions presented in the study are included in the article/Supplementary Material, further inquiries can be directed to the corresponding author.

## Author Contributions

CP and BV contributed to conception and design of the study. CP supervised the experiments. VC, LL, BG, and PD performed the experiments. CP, VC, LL, and MT analyzed the data. VC and MT produced the figures. CP and VC wrote the first draft of the manuscript. CF provided *Candida* strains. BV supervised the study. All authors contributed to manuscript revision, read, and approved the submitted version.

## Conflict of Interest

The authors declare that the research was conducted in the absence of any commercial or financial relationships that could be construed as a potential conflict of interest.

## Publisher’s Note

All claims expressed in this article are solely those of the authors and do not necessarily represent those of their affiliated organizations, or those of the publisher, the editors and the reviewers. Any product that may be evaluated in this article, or claim that may be made by its manufacturer, is not guaranteed or endorsed by the publisher.

## Footnotes

1
https://www.metaboanalyst.ca/
