# Supplementary material for: Lactobacillus Biofilms Influence Anti-Candida Activity
Source: Front Microbiol. 2021 Oct 29;12:750368. doi: 10.3389/fmicb.2021.750368 (PMC8586509; doi:10.3389/fmicb.2021.750368)
Supplement: Supplementary file 1 [file Data_Sheet_1.PDF]

Supplementary Material

Supplementary Figures

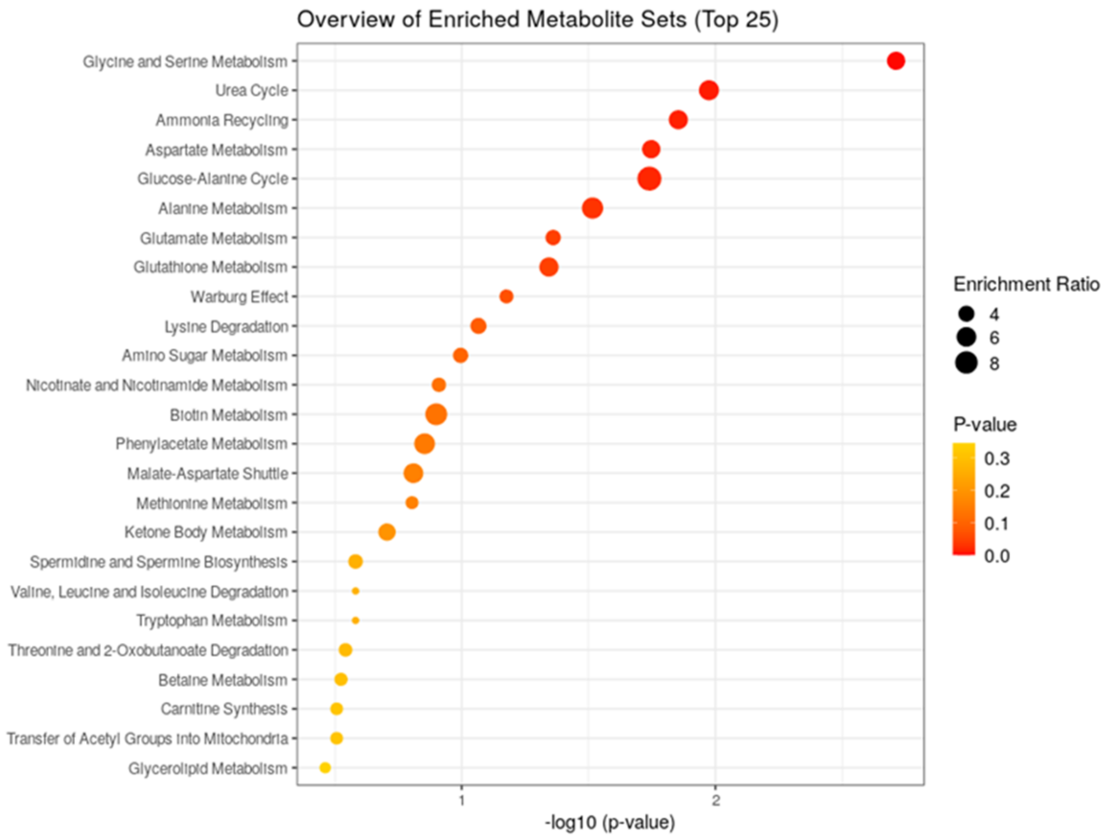

Supplementary Figure S1. The top 25 metabolic pathways analysis of *Lactobacillus* CFS, based on significant different metabolites between bf-CFS and pk-CFS, identified by the enrichment analysis (MetaboAnalyst).

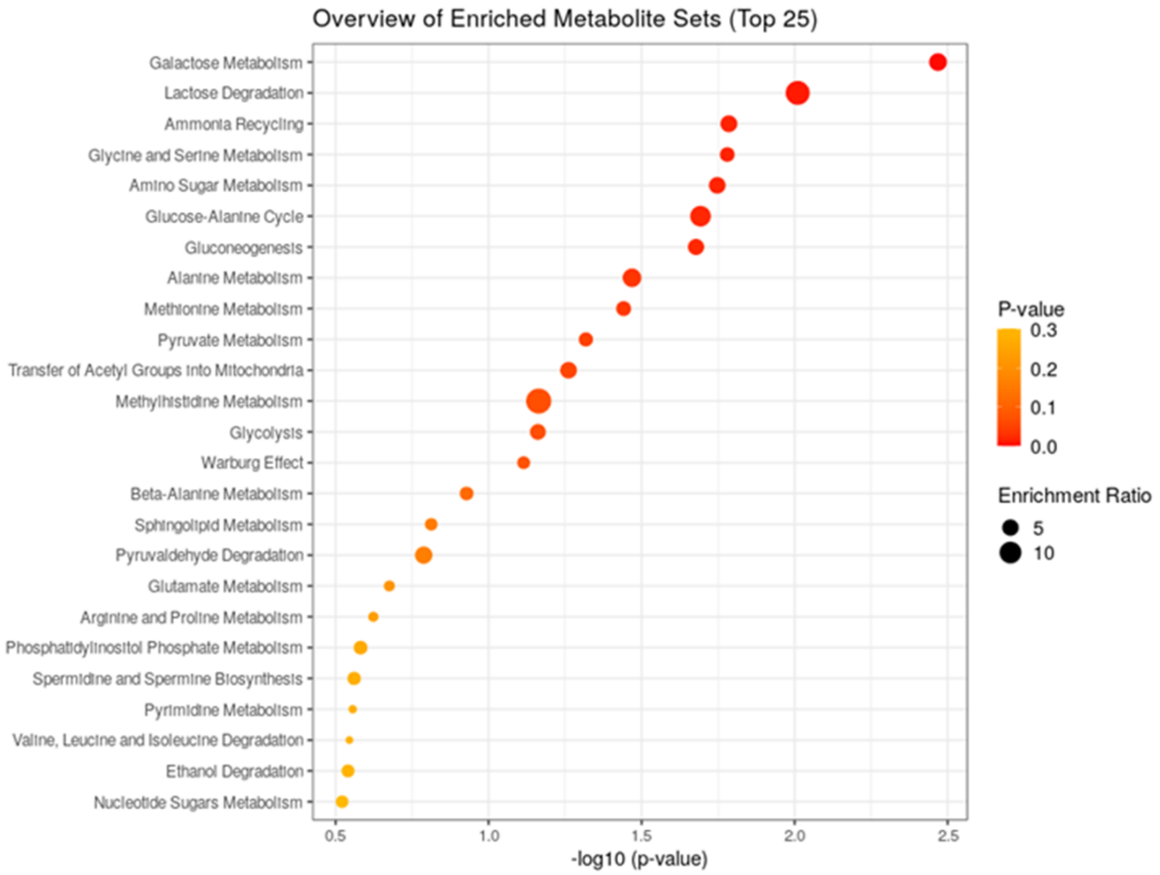

Supplementary Figure S2. The top 25 metabolic pathways analysis of *Lactobacillus* CFS, based on significant different metabolites between high activity CFS and low activity CFS, identified by the enrichment analysis (MetaboAnalyst).
